# Small Bowel Adenocarcinoma Mimicking a Crohn’s Attack

**DOI:** 10.7759/cureus.15743

**Published:** 2021-06-18

**Authors:** Arda Yavuz, Gülnihal Ay, Kübra Akan, Celal Ulaşoğlu, İlyas Tuncer

**Affiliations:** 1 Gastroenterology and Hepatology, Istanbul Medeniyet University Göztepe Research and Training Hospital, Istanbul, TUR; 2 Pathology, Istanbul Medeniyet University Göztepe Research and Training Hospital, Istanbul, TUR

**Keywords:** small bowel adenocarcinoma, crohn’s disease, inflammatory bowel disease, colonoscopy, ileum

## Abstract

Small bowel adenocarcinoma (SBA) is a rare disease and presents with intermittent abdominal pain, weight loss, nausea, vomiting, and gastrointestinal bleeding. In cases with delayed diagnosis, intestinal obstruction or bowel perforation can also be observed. In our case, the patient presented with ileus after an operation that was diagnosed with SBA. After six cycles of chemotherapy, the patient went into complete remission.

## Introduction

Small bowel adenocarcinoma (SBA) is a rare disease, the diagnosis of which remains challenging due to nonspecific symptoms that mimic Crohn's disease (CD). The predisposing diseases include Crohn's disease (8.7%), Lynch syndrome (6.9%), familial adenomatous polyposis (1.7%), celiac disease (1.7%), and Peutz-Jeghers syndrome (0.6%) [1].

Certain vital suggestions in the CD guidelines can help to detect SBA. First, all newly diagnosed patients with CD should undergo small bowel assessments (intestinal ultrasound, magnetic resonance enterography (MR enterography), and/or capsule endoscopy). Second, a patient with suspected clinical CD and a colonoscopy with normal findings should be evaluated with capsule endoscopy. Alternatively, cross-sectional imaging or device-assisted enteroscopy may be performed for endoscopic or histologic confirmation [2]. Moreover, routine surveillance is not recommended.

Herein, we reported a patient with insufficient histological and endoscopic evidence of CD and was treated with mesalazine rather than undergoing further evaluation. One year after diagnosis, she developed ileus and was diagnosed with SBA. We want to emphasize the importance of small bowel assessment, especially for unresponsive treatment and an inefficient diagnostic assessment.

## Case presentation

**Figure 1:**
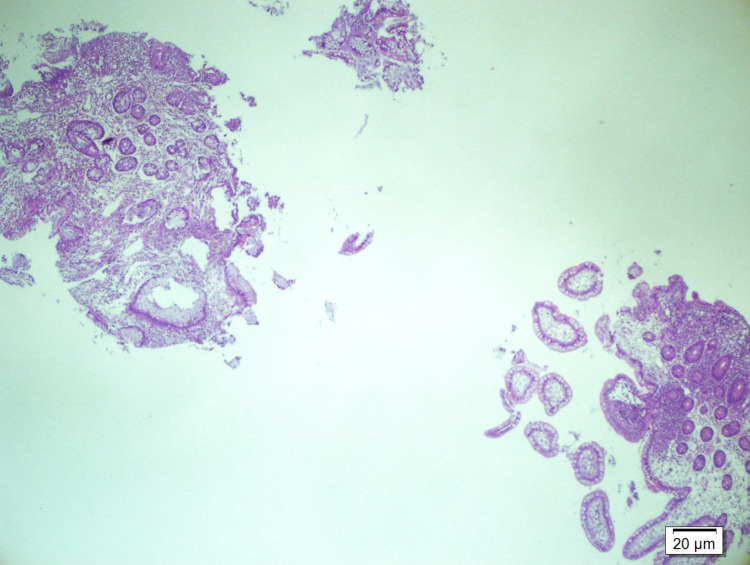
Terminal ileum biopsy: marked architectural distortion and minimal chronic active inflammation (upper left), and normal mucosa (lower right) (HE 40X)

A 34-year-old woman was admitted to our emergency department with stomachache and vomiting. She was treated with mesalazine, as Crohn's disease was suspected for one year, without ulcers on her colonoscopy, and her terminal ileum biopsies were suspicious for inflammatory bowel disease (Figure 1).

**Figure 2:**
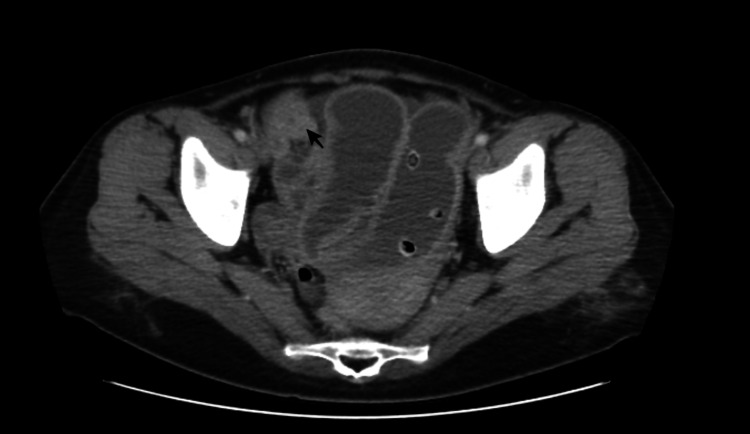
Dilated bowel loops and small bowel carcinoma, which was noticed later (arrow)

It was not clearly understood why the patient with follow-up at an external centre was considered a Crohn's disease. Her vital parameters were normal, and her bowel sounds were hypoactive. Air fluid was observed on imaging, and computed tomography was compatible with the ileus. Thus, a surgeon was consulted, but an operation was not considered at this time. The patient was admitted to the gastroenterology clinic for the subileus. She was followed up without nasogastric drainage because she did not accept nasogastric tube insertion. During the follow-up period, complaints of vomiting and abdominal pain continued, and the patient could not eat entirely. No findings suggestive of paralytic ileus were found in her examinations. After ten days of observation, no clinical response was observed. CT was re-evaluated, a mass image was noticed (Figure 2).

**Figure 3:**
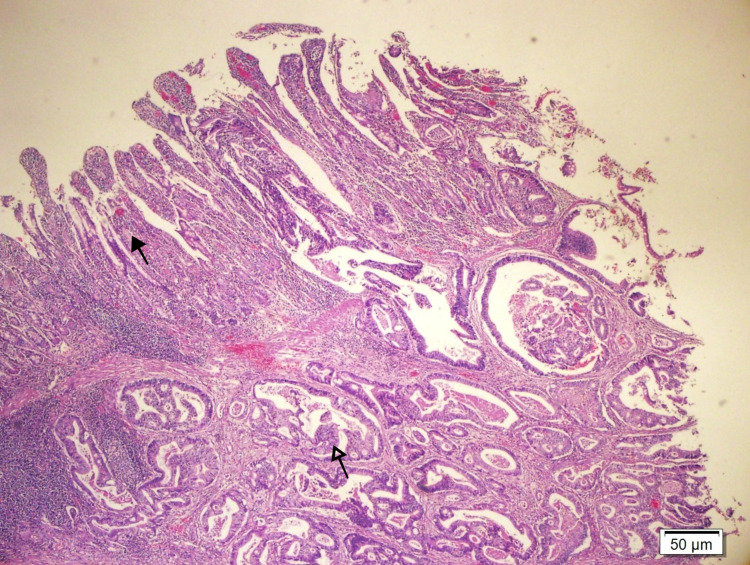
Normal small bowel mucosa (dark arrow) and tumor invasion in mucosa and submucosa (blank arrow) (HE 40X)

**Figure 4:**
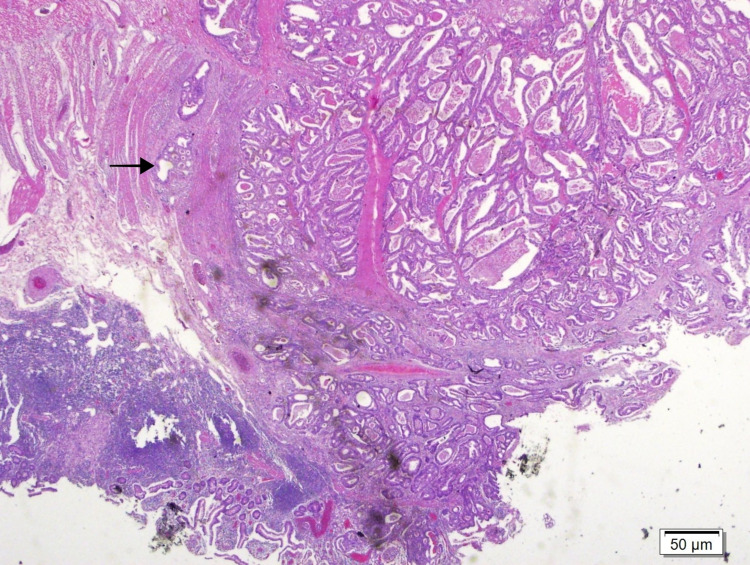
Tumoral invasion in muscularis propria (dark arrow) (HE 20X)

The decision to perform surgery was made. There was a 2cm stricture at a 60cm distance from the ileocecal valve. The diameter of the bowel was 2cm proximal and 5cm distal to the stricture. A 4.5*2.5*1.5cm tumour that invaded the subserosa and lymphovascular invasion was also observed (Figure 3, 4).

**Figure 5:**
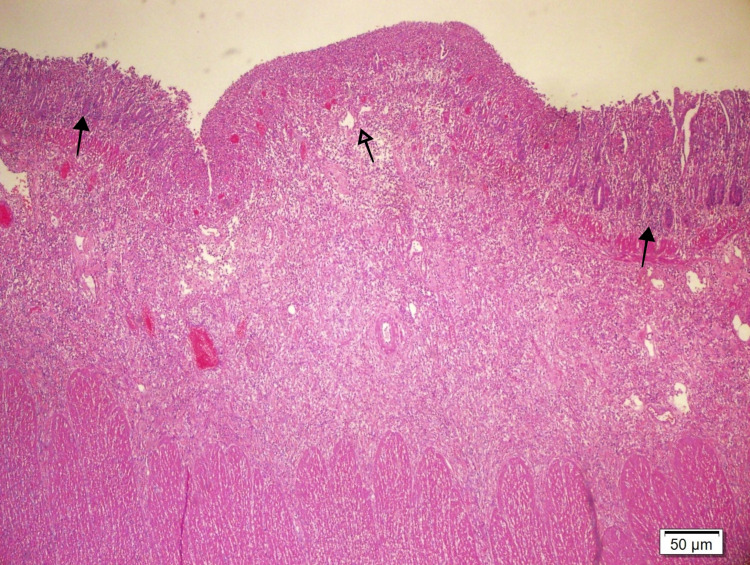
Mucosal and submucosal ulceration (blank arrow) and skip area (dark arrow) proximal to tumor in resection specimen (HE 40X)

The small intestine proximal to the tumour was dilated. Microscopically there was mucosal and submucosal ulceration and skip areas. No granulomata are identified (figure 5).

No loss of mismatch repair (MMR) protein expression was observed on pathological examination. Following surgery, Positron Emission Tomography - Computed Tomography (PET-CT) was performed, and focal metastases in liver segment five and two metastatic implants of 12mm and 13mm in size were observed in the left lower quadrant of the abdomen. From September 2020 to February 2021, she underwent six cycles of chemotherapy with 5-fluorouracil and oxaliplatin. After completion of chemotherapy PET-CT results were normal. Furthermore, in March 2021, a colonoscopy was performed. There was no evidence of Crohn's disease or malignancy.

## Discussion

SBA represents 31%-40% of all small bowel cancer diagnoses. These primary tumors are located in the duodenum (60%), jejunum (25%-29%), and ileum (10%-13%) [3]. These are mostly diagnosed at advanced stages, such as colon carcinoma, and, despite treatment, show poor outcomes. The etiology remains unclear, but certain theories have been proposed [4]. One includes the dilution of digestive material through the small intestine and less irritation at the distal segments. This explains why two-thirds of SBAs arise in the duodenum. Second, the small bowel does not store anything, which explains why the incidence of colon carcinoma is higher than that of SBA. The small intestine plays a major role in the immune system; however, the role of gut microbiota remains unclear.

The relationship between SBA and Crohn's disease remains unclear due to the statistical power of the published studies. In a recent French nationwide cohort study, the incidence of SBA was 0.235 per 1000 patient-years among patients with small bowel CD and 0.464 per 1000 patient-years among those with small-bowel CD lasting for more than eight years. Although there is a high relative risk, the absolute risk of developing small bowel cancer in patients with CD is low [5]. Distal jejunal/ileal CD localization, chronic penetrating disease, long disease duration, young age at diagnosis, male sex, steroids and immunomodulators, small bowel bypass loops, strictures, and environmental factors have been reported as risk factors [6]. However, some studies have failed to confirm these associations. Small-bowel resection and the use of aminosalicylates for more than two years are associated with a lower risk of SBA. Patients with long-standing CD and stricturing disease have the highest risk of developing SBA [7].

Advanced imaging and endoscopic techniques can facilitate the early detection of SBA, but these are not recommended because they are not cost-effective for routine surveillance. A sacculated loop with asymmetrical thickening or benign fibrostenosis-like short-segment stenosis can be seen on computed tomography or magnetic resonance imaging [8]. Capsule endoscopy can detect neoplastic lesions but does not allow for biopsy. Double-balloon enteroscopy or surgery is useful when the patient suffers from complications such as small bowel obstruction or non-responsive small bowel strictures or fistulas.

## Conclusions

We report a case of a patient with SBA that presented with suspected CD but lacked significant diagnostic evidence. Further investigation led to the diagnosis of SBA, which was diagnosed at a late stage. We want to emphasize the importance of early small intestine assessment in patients who are unresponsive to treatment or when CD diagnoses are doubtful.
